# Effect of Fibre Orientation on Impact Damage Resistance of S2/FM94 Glass Fibre Composites for Aerospace Applications: An Experimental Evaluation and Numerical Validation

**DOI:** 10.3390/polym14010095

**Published:** 2021-12-27

**Authors:** Khaled Giasin, Hom N. Dhakal, Carol A. Featheroson, Danil Yurievich Pimenov, Colin Lupton, Chulin Jiang, Antigoni Barouni, Ugur Koklu

**Affiliations:** 1 Advanced Polymers and Composites (APC) Research Group, School of Mechanical and Design Engineering, University of Portsmouth, Anglesea Road, Anglesea Building, Portsmouth PO1 3DJ, UK; hom.dhakal@port.ac.uk (H.N.D.); colin.lupton@port.ac.uk (C.L.); chulin.jiang@port.ac.uk (C.J.); antigoni.barouni@port.ac.uk (A.B.); 2 School of Engineering, Cardiff University, The Parade, Cardiff CF24 3AA, UK; featherstonca@cardiff.ac.uk; 3 Department of Automated Mechanical Engineering, South Ural State University, Lenin Prosp. 76, 454080 Chelyabinsk, Russia; danil_u@rambler.ru; 4 Department of Mechanical Engineering, Faculty of Engineering, Karamanoglu Mehmetbey University, 70100 Karaman, Turkey; ugurkoklu@kmu.edu.tr

**Keywords:** S2/FM94, composites, low-velocity impact, finite element modelling, composite damage, computed tomography

## Abstract

This study aims to investigate the influence of fibre orientation and varied incident energy levels on the impact-induced damage of S2/FM94, a kind of aerospace glass fibre epoxy/composite regularly used in aircraft components and often subjected to low-velocity impact loadings. Effects of varying parameters on the impact resistance behaviour and damage modes are evaluated experimentally and numerically. Laminates fabricated with four different fibre orientations ${\left[0/90/+45/-45\right]}_{8s}$, ${\left[0/90/90/0\right]}_{8s}$, ${\left[+45/-45\right]}_{16s},$ and $\;{\left[0\right]}_{32}$ were impacted using three energy levels. Experimental results showed that plates with unidirectional fibre orientation failed due to shear stresses, while no penetration occurred for the ${\left[0/90/90/0\right]}_{8s}$ and ${\left[+45/-45\right]}_{16s}$ plates due to the energy transfer back to the plate at the point of maximum displacement. The impact energy and resulting damage were modelled using Abaqus/Explicit. The Finite Element (FE) results could accurately predict the maximum impact load on the plates with an accuracy of 0.52% to 13%. The FE model was also able to predict the onset of damage initiation, evolution, and the subsequent reduction of the strength of the impacted laminates. The results obtained on the relationship of fibre geometry and varying incident impact energy on the impact damage modes can provide design guidance of S2/FM94 glass composites for aerospace applications where impact toughness is critical.

## 1. Introduction

The global demand for composites is projected to grow by 173%, reaching $41.4 billion by 2025 from a record $23.8 billion in 2020 [1]. Composite materials can be engineered to achieve enhanced mechanical properties in desired directions. The most common types used are carbon fibre and glass fibre composites. Glass fibre composites have many applications in aerospace, automotive, and marine industries. Glass fibre composites bring weight reductions in aircraft compared to those made entirely from traditional aluminium alloys. Glass fibre composite structures used in aircraft are mainly made from E and S glass fibres due to their superior tensile and compressive strengths. Type S glass fibres are also used in GLARE^®^ fibre metal laminate, which is installed in parts of the fuselage of the Airbus A380 [2]. The first commercial aircraft to contain a composite structure made from glass fibre was the Boeing 707 jet in the 1950s, where it comprised about 2% of the structure. Nowadays, the content of composites in modern commercial aircrafts such as the Boeing 787 Dreamliner and the Airbus A350 XWB has reached just over 50% of their weight. However, composites are prone to various failure modes in the fibre and matrix. For example, delamination between adjacent plies, fibre kinking and breakage, matrix cracking, and debonding are the main failure modes. From those damages reported earlier, delamination is the most critical form of damage that occurs in laminates subjected to low-velocity impact due to the weak performance of fibres to the overall strength of the composite in the impact direction, especially for thin laminates [3,4]. Indeed, composite structures tend to have weak resistance to impact damage by foreign objects [5]. Damage caused by impact can affect the load-carrying capacity, particularly when the structure is under compression [5]. Damage in aeronautical composite structures may occur during the taking off and landing of the aircraft due to high-velocity impact from bird strike, metal fragments, hailstone etc. It can also occur due to low-velocity impact during aircraft ground service from accidental falling objects such as hammers, boxes etc. The severity of the impact damage on composite structures can vary from full penetration to barely visible impact damage. The latter can be critical for the safety and integrity of the aircraft structure since visual inspection will not detect subsurface damage. The definition of a low-velocity impact itself is based on the speed of the falling weight or the level of damage on the structure, as reported by several researchers in the past [3,6,7,8]. Some researchers considered a low impact test is that of which the speed of the falling object was less 10 m/s [3,7]. In another definition, it was that which occurs at impact speeds below 100 m/s [3,6]. The damage in composites due to low-velocity impacts can be critical at the micro-level as it could lead to a severe reduction in the material post-impact residual strength and stiffness [3]. Therefore, studying impact damage is important, especially with modern aircraft, which have an increasing percentage of composites in their structures. There are four main failure mechanisms (failure modes) that occur in fibre reinforced composites due to low-velocity impact loading [9]. The first failure mechanism occurs in the matrix due to tension, compression, or shear loading. The matrix failure mode results in cracking parallel to the fibres and debonding between the fibres and the matrix. The main reason for these failures is related to the property mismatch between the composite constituents (i.e., the fibre and the matrix). The second failure mechanism occurs in fibres subjected to tensile (fibre breakage) or compression (fibre buckling) loading. The third failure mechanism is due to the interlaminar stresses which are responsible for delamination. The fourth failure mechanism occurs when the impactor fully perforates the laminate, a phenomenon that is more common at the ballistic impact range. Core buckling and shearing can be considered as a fifth failure mechanism, but this occurs in composite sandwich structures [10]. Nevertheless, previous studies reported that perforation damage in low velocity impact loading is mainly affected by the laminate thickness for CFRP laminates and by glass fibre treatment in GFRP laminates [11]. There are many studies which devoted their efforts to study the low-velocity impact behaviour of E-glass and carbon fibres. However, only a handful of studies can be found in the open literature which investigated the damage in S2 glass fibre composites due to low velocity impact, an essential prerequisite to increase the use of S2 glass fibre composites in industry. The ability of the fibre to store energy elastically is of great importance. According to Satishkumar et al. [12], S2 glass fibres have the highest young’s modulus, tensile strength, and percentage elongation at break, among many other types of glass fibres such as (A, C, D, E, R, EGR, and AR). This means that fibres with a higher modulus of elasticity and failure strain can better resist damage due to low-velocity impact loading and absorb higher elastic energy [11].

The impact damage in composite laminates is governed by the type of the fibre, its orientation, matrix properties, sample thickness, impact velocity, and geometrical characteristics of the impacting projectile and service condition [13]. An important factor that can influence the damage tolerance and durability in a composite structure is the stacking sequence of the laminate. Indeed, the fibres in a composite can carry between 70 to 90% of the load applied to the composites structure [14]. The relationship between the ply orientation in a composite and the impact damage resistance is rather complex due to the multidirectional behaviour of the composite and the way the damage propagates within the laminate. The adhesion (bonding) strength and quality within the fibre/matrix system of the composites can also play an important role in impact damage resistance [15]. Early studies showed that altering the stacking sequence in a composite can influence the impact damage in a greater way than altering its thickness [9]. Belingardi and Vadori [16] reported that glass fibre composites with a stacking sequence of [0/90] exhibit the highest saturation energy (i.e., better impact resistance) compared to the other tested fibre orientations ${\left[0/+60/-60\right]}_{s}$, ${\left[0/+45/-45\right]}_{s}$. Moreover, Sikarwar et al. [17] studied the influence of thickness and fibre orientation on the impact response of woven glass fibre composites. They found that [0/90] laminates showed the best impact resistance among all tested lay-ups which was mainly related to the failure strain which is highly influenced by the fibre orientation in the laminate. Evci et al. [3,18] reported that woven composites showed better impact resistance than unidirectional composites due to their higher ultimate tensile strength. Karakuzu et al. [19] found that the mass of the impactor will increase the impact velocity and that the absorption capability of E-glass fibre plates subjected to impact is more influenced by the mass of the impactor rather than the impact velocity. In addition, Mitrevski et al. [20] demonstrated the effect of the impactor shape on the resulting damage and damage mechanisms during the impact of woven composite laminates. Their results showed that hemispherical impactors gave the largest damage area and minimal for conical shaped impactors. Icten et al. [21] investigated the influence of low temperatures on the impact behaviour of quasi-isotropic glass/epoxy composite plates. They found that damage level was similar regardless of the temperature when using low impact energy and becomes significantly different at higher energy levels. Quaresimin et al. [22] also found that the thickness and the fibre orientation in the laminate can influence its impact energy absorption capability such that using an [0/45] interface showed the least damage due to impact.

The finite element method combined with the impact test became a popular technique to support the evaluation of the damage response and behaviour in composites. There is various published work that investigated the low-velocity impact of GFRP (glass fibre reinforced composites). Yarn-level finite element models combined with the experimental method were applied to analyse the damage behaviour and failure mode for carbon/glass hybridization through a low-velocity impact test [23]. A continuum damage mechanical model was developed and validated to present the effects of carbon/glass/basalt hybridization and fabric structure on the low-velocity impact resistance under different impact energy levels [13]. The low-velocity impact of glass fibre-reinforced polyamide was compared through experiment and FE to confirm that the impact force increased with increasing fibre volume fractions [24]. In another study, the projectile shape, size, and striking location were investigated experimentally and numerically through low-velocity impact loading [25]. A. Kumar Kaviti et al. used numerical analysis of dynamic low-velocity impacts to analyse the elastic behaviour of E-Glass and Carbon/Epoxy composites made with different stacking sequences and found that the material properties largely influence the impact dynamics [26]. However, 90% of composites produced are reinforced with glass fibres [27]. E-glass is the most commonly used glass in composites and dominates the majority of the work published on low-velocity impact in the open literature. There are not many reported works analysing the impact damage behaviour of S2-glass fibre reinforced composites using both experimental and numerical techniques. Therefore, the current study aims to fill this gap in the literature by carrying out systematic experimental tests for S2-glass fibre plates and validated them using numerical modelling. The composite plates are fabricated with different fibre orientations to assess the severity of visible and subsurface damage that occurs due to the change in fibre direction subjected to low-velocity impact conditions using different energy levels (impact velocities). In addition, impact simulation was performed using Abaqus/Simulia FE software using 2D continuum shell elements to model the failure modes in the fibre and the matrix. The FE model was used to simulate the impact process and predict the behaviour of the different plates at different impact energies used in this study.

## 2. Materials and Methods

### 2.1. Composite Plates Manufacturing and Sample Preparation

**Figure 1:**
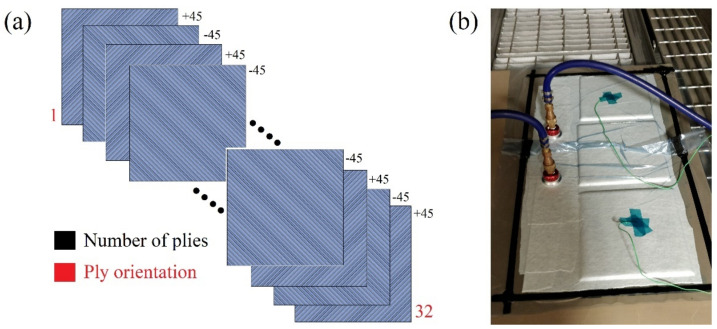
(**a**) Ply configuration for one of the glass fibre plates used in the study ${\left[+45/-45\right]}_{16s}$ (**b**) Manufacturing setup of the workpiece inside the autoclave.

Four plates of S2/FM94 glass fibre, each having dimensions of 240 mm × 240 mm × 4.256 mm thick (thickness is based on 0.133 mm ply thickness with a nominal fibre volume fraction of 59% and a total of 32 plies in each plate) [28], were used in the impact tests as shown in Figure 1.

Large panels were manually laid up from FM-94 adhesive film embedded with S2 glass fibre in the form of prepregs roll manufactured by Cytec^®^ Industrial Materials Limited^®^, Heanor, U.K [29,30,31,32,33]. The panels were then cut into nine square plates (70 mm × 70 mm each) to be used in the impact tests according to BS EN ISO 6603-1:2000. The BS EN ISO 6603-1:2000 standard states that the samples should be squared, have a minimum size of 60 mm, should be placed on the top of a 40 ± 2 mm punctured hole clamping system, and be impacted using a 20 mm diameter spherical striker (impactor). The prepregs were stacked in different arrangements to achieve the desired fibre orientations in each plate, as shown in Table 1. The large panels were cured in an autoclave for 5 h at elevated temperatures of 120 °C and under a pressure of six bars according to the supplier guidelines [28]. The designated prepreg orientation employed in the plates was to mimic specific aircraft structures based on those used in standard grades of GLARE^®^ laminates due to the main beneficial characteristics which those orientations provide, such as fatigue and strength [0/0], impact [0/90/90/0], shear and off-axis properties [+45/−45] [29]. Moreover, The general manufacturing process of composite material components restricts the stacking sequence combination to laminates with 90°, ±45°, 0° oriented plies [34].

### 2.2. Setup of Impact Machine and Test Parameters

**Figure 2:**
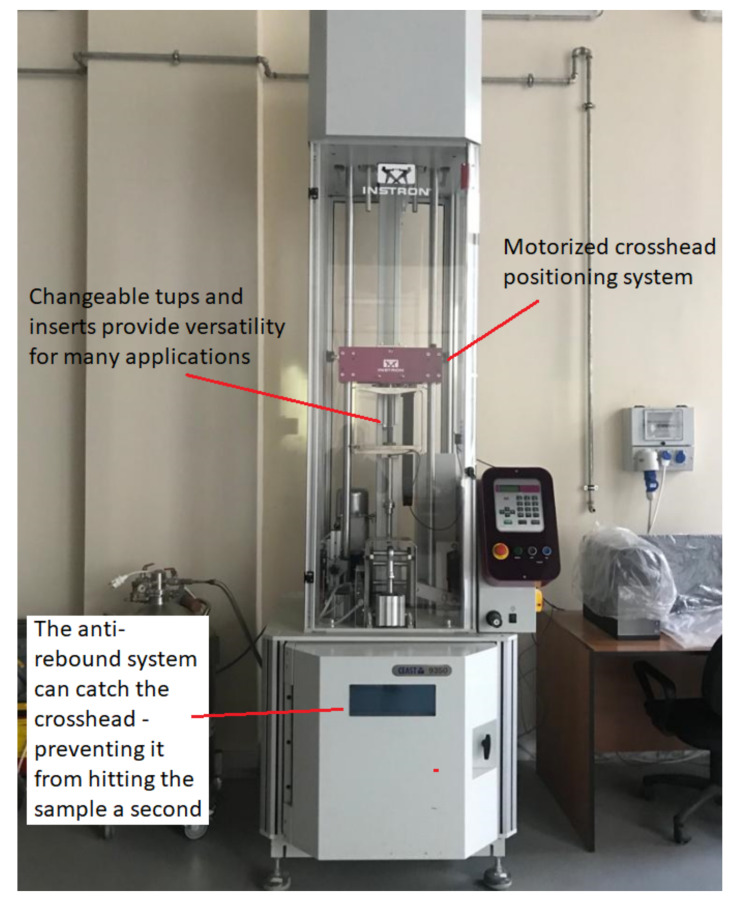
CEAST 9350 drop-tower impact system used in the current study.

The impact tests were carried out on an Instron CEAST 9350 drop-tower impact system (Instron, Norwood, MA, USA), as shown in Figure 2. The CEAST 9350 is equipped with a motorized crosshead positioning system and data acquisition to simplify the analysis. CEAST VisualIMPACT software was used to control the drop-tower pendulums, set up the test parameters, and for data acquisition during the impact tests. The software can record the impact force and absorb energy data related to impact. The drop-tower impact system has an energy range of 0.59–1800 J and impact velocities between 0.77–24 m/s. The range of the drop height is between 0.03 and 29.4 m. The drop weight used in the impact tests can be varied depending on the impact parameters using interchangeable falling masses between 2.00 and 70.0 kg. Clamping plates for the CEAST 9350 machine use a pneumatic system to push the sample against the test stand, holding it securely in place during the impact test. The clamping plates recommended for rigid plastics on CEAST 9350 were used, which were designed according to ISO 6603-1 testing standards and had a clamping diameter of 40 mm. The impact tests were conducted using a 20 mm diameter hemispherical tup.

The impact energies used in the tests are provided in Table 2. All tests performed in this study are classified as low-velocity impact tests, with the initial velocity being 4.44 m/s (i.e., for velocities < 10 m/s low-velocity impact is considered) [35]. Each set of plates with a specific ply orientation was impacted using the same energy level three times to confirm the tests were repeatable. Therefore, all the energy vs. time and energy vs. displacement data reported thereafter represent the mean values of the three repetitions, which give a total number of 36 impacted samples. The levels of impact energy used in the study were selected according to past studies reported previously on the impact of glass fibre composites.

### 2.3. Computerised Tomography

**Figure 3:**
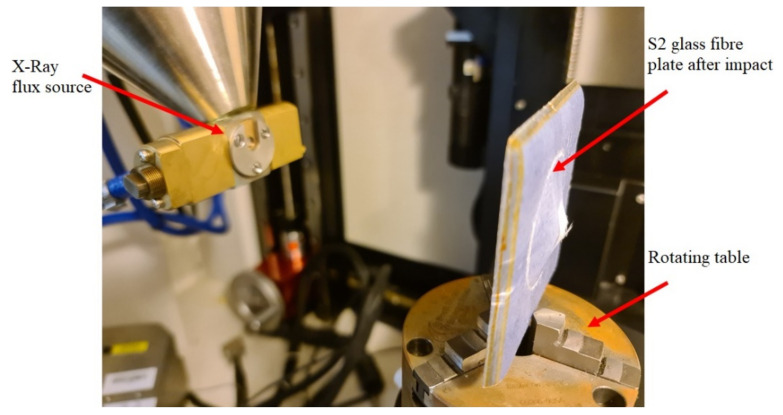
CT scan of glass fibre plates after impact.

X-ray micro-CT scanning (computed tomography) was used to observe the different forms of damage and the presence of delamination, which might have occurred within the impacted plates [3]. A Nikon XT H 225 X-ray machine CT scan machine (Minato, Tokyo, Japan) was used in this study. The setup of the plate inside the CT scanner is shown in Figure 3. The CT scanner operates on a 225 KV/2000 mA voltage-current and can achieve a minimal focal spot size of 3 µm. In the current study, the resolution was set to 164 µm to enable capturing the whole plate while it rotates 360° inside the machine, which resulted in a total of 2400 projections. The projections were then processed to generate the 3D volume of the plate using Volume Graphics VG Studios Max version 2.0. The maximum resolution obtained for the scanned plates was 492 × 492 × 492 pixels.

## 3. Numerical Model

**Figure 4:**
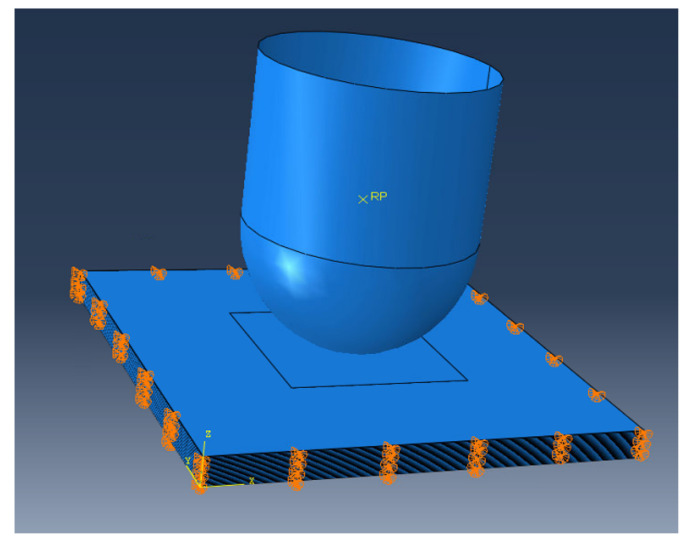
FE model of the composite plate impact.

A numerical model was developed using Abaqus FE software to simulate the impact process and predict the behaviour and the onset and propagation of the damage in the tested plates. The FE model comprised of the impactor and the plate, as shown in Figure 4. The impactor was modelled as a discrete rigid body since its deformations are not of interest during the impact phenomenon. The mass and inertia of the impactor were calculated based on the experimental data and the energy level applied on each experimental set. The motion of the impactor was prescribed by applying boundary and initial conditions at its reference point. The impactor translational motion was constrained in X and Y directions and was free to move in the Z direction, while the rotational motion was constrained in all directions. The initial condition for the velocity of the impactor matched the velocity measured during the experiment at 4.4 m/s. The impactor interacts with the plate through the general contact algorithm using a penalty enforcement contact method and a friction coefficient of µ = 0.3 [36].

### 3.1. Intralaminar Damage Model

Prior to damage initiation, material behaviour is assumed to be linearly elastic [37]. After that, the FE model is capable of predicting the damage initiation both in the matrix and the fibres based on the 2D Hashin criterion, which is applied on a ply level using the built-in definition available in the software [37,38]. The detailed description of the Hashin criterion is reported in Abaqus documentation [37]. The Hashin damage initiation criteria are based on four different failure modes for fibre and matrix failure in compression and tension modes. The damage initiation defines the point of initiation of degradation of stiffness. Equation (1) below shows the four damage modes in Hashin’s damage initiation criteria.


(1) $$\begin{array}{ccc} \mathrm{Fibre}\;\mathrm{tensile}\;\mathrm{failure}\;\mathrm{mode}\\ \left(\sigma_{11}\geq 0\right) & F_{f}^{t}={\left(\frac{\sigma_{11}}{{Χ}_{Τ}}\right)}^{2}+a{\left(\frac{\sigma_{12}}{S_{L}}\right)}^{2} & \\ \mathrm{Fibre}\;\mathrm{compression}\;\mathrm{failure}\;\mathrm{mode}\\ \left(\sigma_{11}<0\right) & F_{f}^{c}={\left(\frac{\sigma_{11}}{{Χ}_{C}}\right)}^{2} & \\ \mathrm{Fibre}\;\mathrm{tensile}\;\mathrm{failure}\;\mathrm{mode}\\ \left(\sigma_{22}\geq 0\right) & F_{m}^{t}={\left(\frac{\sigma_{22}}{Y_{T}}\right)}^{2}+{\left(\frac{\sigma_{12}}{S_{L}}\right)}^{2} & \\ \mathrm{Matrix}\;\mathrm{compression}\;\mathrm{failure}\;\mathrm{mode}\\ \left(\sigma_{22}<0\right) & F_{m}^{c}={\left(\frac{\sigma_{22}}{2S_{Τ}}\right)}^{2}+\left[{\left(\frac{Y_{C}}{2S_{T}}\right)}^{2}-1\right]\frac{\sigma_{22}}{Y_{C}}+{\left(\frac{\sigma_{12}}{S_{L}}\right)}^{2} \end{array}$$

In the equations above, $X_{T}$, $X_{C}$, $Y_{T}$, and $Y_{C}$ are the tensile and compression strengths in the longitudinal and transverse fibre directions. $S_{L}$ and $S_{T}$ are the shear strength parameters in the 12 and 13 planes. The Hashin’s damage data for ply strengths are given in Table 3 [39,40,41].

Damage evolution occurs after one of the damage initiation criteria is satisfied. It describes the rate of degradation of material stiffness which leads to material failure [37]. The material behaviour post-damage initiation is given in the form of $\sigma =C\left(d\right):\varepsilon ,\;$where *C*(*d*) is the damaged elasticity matrix shown in Equation (2) below:

(2) $$C\left(d\right)=\frac{1}{D}\left[\begin{array}{ccc} \left(1-d_{f}\right)E_{1} & \left(1-d_{f}\right)\left(1-d_{m}\right)v_{21}E_{1} & 0\\ \left(1-d_{f}\right)\left(1-d_{m}\right)v_{12}E_{2} & \left(1-d_{m}\right)E_{2} & 0\\ 0 & 0 & \left(1-d_{s}\right)G \end{array}\right]\;D=1-\left(1-d_{f}\right)\left(1-d_{m}\right)v_{12}v_{21}>0\;d_{s}=1-\left(1-d_{ft}\right)\left(1-d_{fc}\right)\left(1-d_{mt}\right)\left(1-d_{mc}\right)$$

The damage variables $d_{f}$,$\;d_{m}$, and $d_{s}$ are the current state of fibre, matrix, and shear damage, respectively, that are derived from four damage variables $d_{ft}\;,d_{fc}\;,d_{mc},\;d_{mc}$ which describe damage for each failure mode. The damage was evaluated at each integration point at every step of the simulation [37]. The material properties of S2 glass fibre used in FE analysis were obtained from past literature and are given in Table 4 [39,40,41]. Where *E*_11_, *E*_22_, *E*_33_, *G*_12_, *G*_13_, and *G*_23_ are the elastic and shear modulus in the fibre and transverse directions, respectively, and *ν*_12_, *ν*_13_, and *ν*_23_ are Poisson’s ratios in plane 1–2, 2–3 and 1–3, respectively [41].

An energy evolution law with linear softening behaviour was used to model the energy dissipation during the damage process and consequently the removal of elements from the mesh upon complete failure and consequent removal of the failed elements (erosion). The fracture energies used to calculate the damage evolution are shown in Table 5 [42]. Where $G_{ft}^{C}$ and $G_{fc}^{C}$ are the longitudinal tensile and compressive fracture energies. $G_{mt}^{C}$ and $G_{mc}^{C}$ are transverse tensile and compressive fracture energies.

### 3.2. Interlaminar Damage Model at Interfaces

The interlaminar damage and delamination at interfaces can be modelled using the cohesive behaviour in terms of a traction separation law; the law initially assumes a linear elastic behaviour prior to damage initiation, after which the failure at the interface is characterized by progressive degradation of the material stiffness [37]. A bi-linear traction-separation energy-based law was used to model the damage evolution and degradation of bonding interfaces, where the damage initiation was predicted using the quadratic nominal stress criterion [37], as shown in Equation (3). In this criterion, the onset of damage occurs when a quadratic interaction function involving the nominal stress ratios reaches a value of one.

(3) $${\left\{\frac{\langle t_{n}\rangle }{t_{n}^{0}}\right\}}^{2}+{\left\{\frac{t_{s}}{t_{s}^{0}}\right\}}^{2}+{\left\{\frac{t_{t}}{t_{t}^{0}}\right\}}^{2}=1$$

In the above equation, $t_{n}$, $t_{s},$ and $t_{t}$ are the nominal traction stress vectors along the local 1,2 and 3-directions, respectively. $t_{n}^{0}$, $t_{n}^{0}$ and $t_{s}^{0}$, $t_{t}^{0}$ are the peak values of the nominal stresses when the deformation is purely normal, purely in the first direction and purely in the second direction to the interface, respectively. The elastic behaviour, inter-laminar strength and fracture toughness parameters for cohesive elements are given in Table 6 [42]. Where *E*/*E_nn_*, *G*_1_/*E_ss,_* and *G*_2_/*E_tt_* normalised elastic and shear modulus, and $G_{n}$, $G_{s}$, $G_{t}$ are the normal, shear (first and second directions) fracture energy modes. Finally, the linear degradation law function of the dissipated energy was used along with the Benzeggagh–Kenane (BK) law for the mixed opening mode [43].

### 3.3. Numerical Implementation

The geometry of the impactor and the glass fibre plate was designed in Abaqus. In the developed model, each composite layer was modelled by modelling the composite ply and the cohesive interface separately. The composite plies were modelled using linear hexahedral elements of type SC8R, whereas linear hexahedral elements of type COH3D8 were used for all the interfaces between the oriented composite plies. The elastic stiffness of the impactor is significantly higher than the glass fibre plate; therefore, it was modelled as a shell 3D discrete rigid and meshed using quadrilateral and triangular elements of type R3D4 and R3D3. Enhanced hourglass control and distortion control options were selected for all the elements in the plate [37]. The meshing details of the impactor and the plate are given in Table 7. To ensure that the impact simulation is not affected by changing the size of the mesh, a mesh convergence analysis was carried out to determine the maximum element size, after which decreasing it further would only result in a slight improvement in the model accuracy. The meshing strategy of the plate was to apply a finer mesh in the vicinity of the impact zone where the impactor will be in contact with the plate and coarser mesh away from the impact zone. The size of the cohesive elements was half the size of the shell elements used for the rest of the laminate. In this case, the model was able to achieve a better representation of the delamination developed in the structure. One element was used through the thickness direction of the cohesive layers. After the solution of the model using the Abaqus/Explicit solver, the energy balance was checked, verifying the correctness of the numerical simulation.

## 4. Results and Discussion

### 4.1. Load Time Response

**Figure 5:**
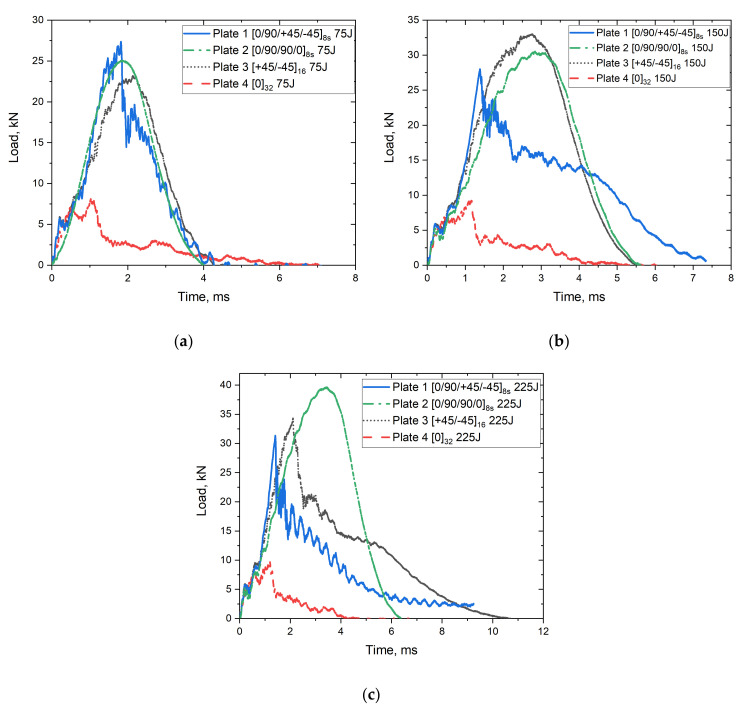
Load vs. time curves for S2/FM94 glass fibre plates with different fibre orientation systems under (**a**) 75 J, (**b**) 150 J, and (**c**) 225 J impact energy.

Figure 5 shows the load vs. time curves resulting from the impact of the plates with different fibre orientations under different impact energies (75, 150, 225 J). The progressive damage is described by the oscillation in the curve from the loading and unloading of the impactor. A smoother curve indicates a lower damage severity in the plate upon impact. According to Figure 5, the curve of incident impact for Plate 2 is smoother than that for other plates, especially when the impact is carried out at 225 J. Thus, the resultant damage from incident impact for Plate 2 is less severe while the resultant damage from the incident impact for Plate 4 is more severe as more oscillations appear in the curves.

**Figure 6:**
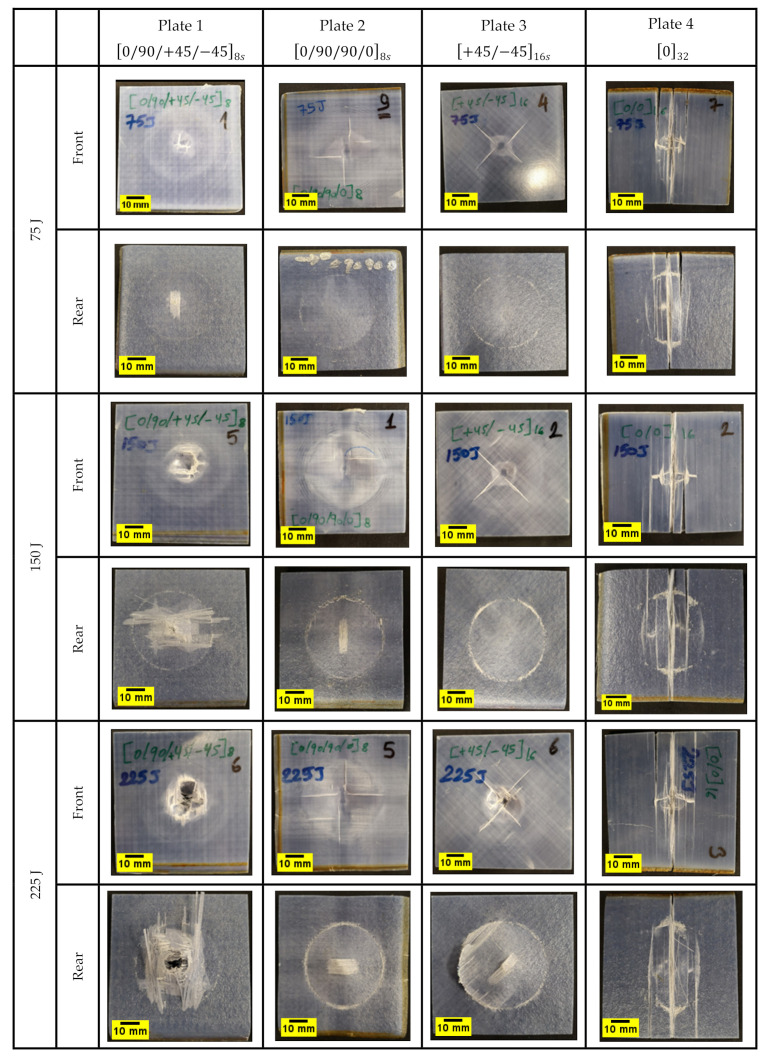
Front and rear images of the plates showing the resulting damage after impact.

This can be confirmed from Figure 6, where images of the specimens after impact are depicted both in their front and rear surfaces. It depicts that the peak contact force is much lower for Plate 4 ${\left[0\right]}_{32}$ then the others, which indicates that applying different fibre orientations can improve the resistance during impact. This may be due to the variation in the stacking sequence of the other plates, which increases the resistance during an impact failure and a unidirectional fabric with the same fibre orientation throughout the plate thickness. Furthermore, Plates 1, 2, and 3 offer similar peak contact force, especially for the 75 J impact energy. Although Plate 4 exhibits a prolonged plateau of energy absorption because the samples are close to penetration, it provides much lower strength and stiffness than others. Other S2/FM94 glass fibre plates with different orientation systems exhibit a higher load-bearing capability. Therefore, introducing different fibre orientations can improve the strength and stiffness with constant density [9,16].

**Figure 7:**
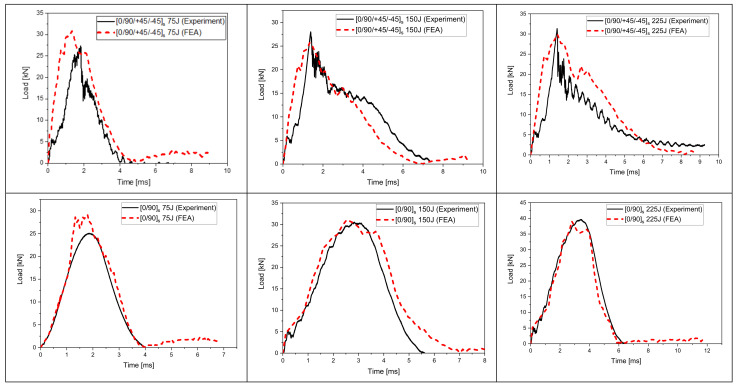

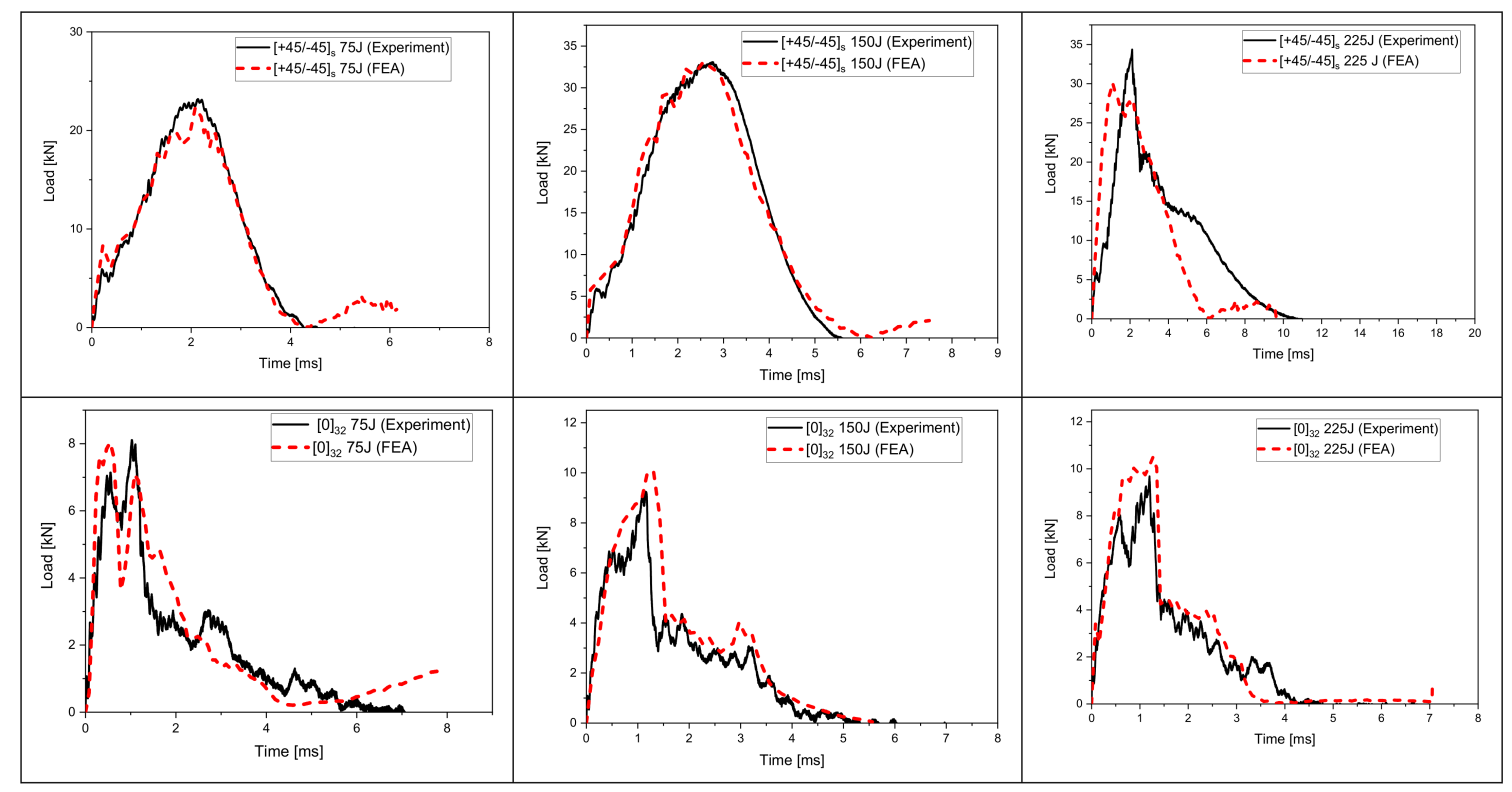
Comparison of the load-time curves between the experimental and FE analysis results.

This is further supported by the FE model which was used to simulate the impact process and resulting damage in the S2/FM94 composite plates. In Figure 7, the experimental load vs. time curves is compared with the FE model for each of the four different plates and each impact energy. It is evident that the FE model has predicted the maximum impact load and the load vs. time profile on the plates for all the impact energies. The model could predict the onset of damage initiation, evolution, and degradation, as well as the consequent reduction of the impact strength of the specimens. The FE results showed similar trends in load vs. time profiles when compared to the experimental data. For example, the results for the ${\left[0/90/90/0\right]}_{8s}$ plate and an impact energy of 150 J, the maximum load from the tests was ~30.5 KN, while the predicted maximum load from the FE model was found to be ~30 KN. Similarly. The results for the ${\left[0/90/90/0\right]}_{8s}\;$plate and an impact energy of 75 J, the maximum load from the tests was ~25 KN. In contrast, the predicted maximum load from the FE model was found to be ~29 KN, which indicates that the FE model can estimate the maximum load with 0.52% and 13% deviation from the experimental test results. However, it was observed that this discrepancy in FE predictions was significant at higher impact energies and for plates with different fibre orientations, such as ${\left[0/90/+45/-45\right]}_{8s}$. This could be due to the inadequacies of the element–deletion criteria used in the 2D-Hashin material damage model [37]. The use of a 3D material damage model allows for a better representation of the through-thickness stress variation in the plies [44]. The discrepancy could be attributed to the variability in the material properties, quality of manufactured plates, and the presence of some imperfections which were introduced during the manufacturing process [45]. Moreover, the behaviour of the material prior to damage initiation was assumed to be linearly elastic. This might have affected the FE estimations since non-linearities were not taken considered in this study (i.e., nonlinear shear stress–strain behaviour) [46]. The accuracy of the FE models can be further improved by using more realistic friction models at the impactor–plate interface which takes into account the variation in the coefficient of friction with impact energy and ply orientation. In addition, the type of elements used can affect the material stiffness and the way the material is deformed [41]. In addition, the effect of the impactor on the impacted plates was neglected as it was modelled as a discrete rigid body. These issues will be addressed in future research work.

### 4.2. Load Displacement Response

**Figure 8:**
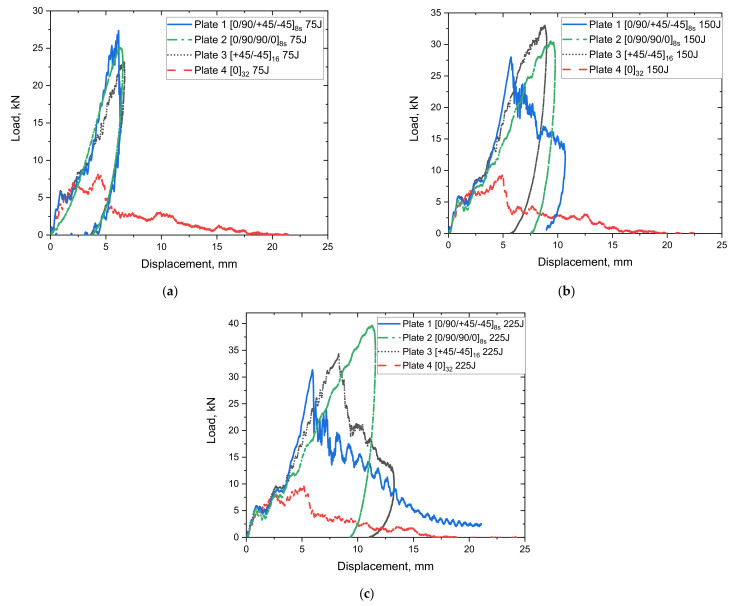
Load–displacement curves for S2/FM94 glass fibre plates with different fibre orientation systems under (**a**) 75 J, (**b**) 150 J, and (**c**) 225 J impact energy.

The load vs. displacement results of the impacted plates at 75 J, 150 J, and 225 J are depicted in Figure 8. The force–displacement curves exhibit some closed contours (closed-loop), and the area under the closed-loop refers to the energy absorbed during the impact. In a closed curve, the ascending part describes the impact bending stiffness, while the descending one stands for lack of penetration during the impact event. According to the load–displacement curves for all the plates under different impact energies, it can be concluded that Plate 1 started to penetrate from 225 J, Plate 2 and 3 did not experience any penetration, and Plate 4 is close to penetration with 75 J. This can also be confirmed by observing Figure 6 (images after impact). For the specimen without any penetration such as Plate 2, the incident energy is fully transferred back to the specimen at the point of maximum displacement [47]. The load–displacement curve illustrates that Plate 2 specimens with greater resistance exhibit enhanced load-bearing capabilities compared to other plates with different fibre orientation systems. Moreover, the comparison of force-displacement curves indicates the influence of ply orientation. In the case of cross-ply and angle-ply laminates, a smoother course of the curves can be observed, while oscillations can be seen in the quasi-isotropic laminate (Plate 1) during the unloading stage. The oscillations suggest possible failures in the laminate due to the decrease in the structural stiffness (near the impact point) [48]. It can also be seen in Figure 8a that at 75 J, quasi-isotropic laminate outperforms cross-ply and angle-ply laminates. However, at higher energies of 150 J and 225 J, there is a divergence in the response of the quasi-isotropic laminate. The cross-ply and angle-ply laminates are characterized by increased stiffness in thickness direction which indicates higher abilities to carry dynamic loads, thus leading to smaller displacement than in quasi-isotropic laminate at higher impact energies can be observed in Figure 8b,c. The work reported by Papa et al. [49] on E-glass laminates with a plate thickness of 3–6 mm and impact energy of 250 J, using the same impactor used in this study, shows that the maximum displacement in E-glass fibre occurs at much lower impact loading (10–23 KN). Meanwhile, in the current study, according to Figure 8c, the maximum displacement is relatively lower than those reported in their study. However, a like-to-like comparison cannot be made due to the differences in experimental setup and material thickness. A firm conclusion can be made that S2 glass fibres have a higher ability to store energy elastically due to their higher young modulus and percentage elongation at break [12]. According to Caminero et al. [50], low-velocity impact of CFRP laminates showed that stacking sequence plays an important role in the impact damage response of the laminate. Their results showed that under the same impact energy level, quasi-isotropic laminates showed slightly higher peak force than cross-ply and angle-ply laminates. However, in the current study, this was true when the impact energy was 75 J. Applying higher impact energies of 150 J and 225 J showed that the peak force in quasi-isotropic laminates is lower than that found in cross-ply and angle ply laminates. This could mean that in S2 glass fibre laminates, there are other factors in addition to bending stiffness that have an influence on the peak load when increasing the impact energy.

### 4.3. Absorbed Energy Time Response

**Figure 9:**
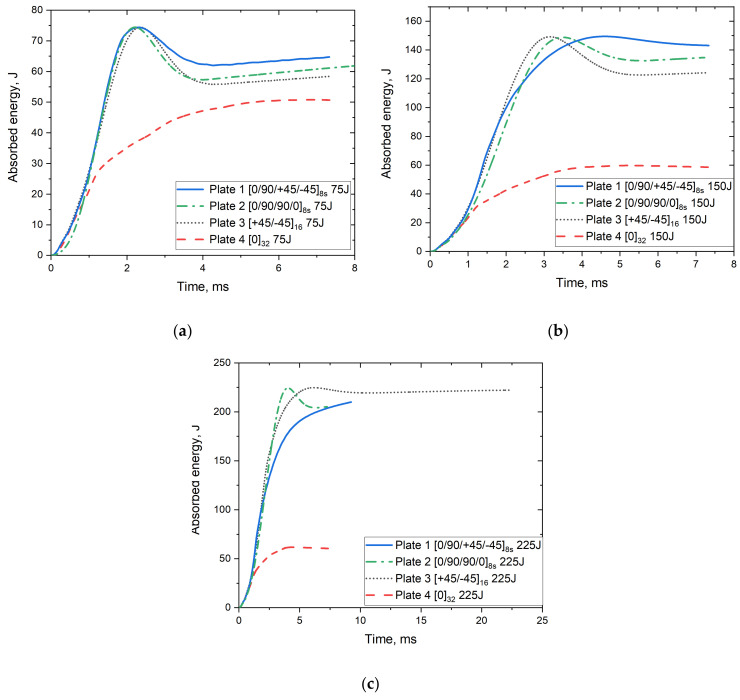
Absorbed energy vs. time curves for S2/FM94 glass fibre plates with different fibre orientation systems under (**a**) 75 J, (**b**) 150 J and (**c**) 225 J impact energy.

The history of the mechanical energy calculated from integrating the force–displacement data is presented in Figure 9. The absorbed energy of the plates increases to the set impact energy and then shows a slight decline until it reaches a constant value of energy with the increase of impact time. The reduction in the absorbed energy is due to the elastic potential energy of the specimens being converted into impactor kinetic energy [51]. It shows that there is some energy dissipation and that not all the energy transferred to the plate is returned to the impactor [52]. Although Plate 4 absorbed much of the available energy, it can only absorb up to 70 J when impacted with 225 J energy due to complete failure. According to Caminero et al. [50], low-velocity impact of CFRP laminates showed that stacking sequence plays an important role in the impact damage response of the laminate. Their results showed that under the same impact energy level, quasi-isotropic laminates showed lower absorbed energy than cross-ply and angle ply laminates. However, in the current study, this was true when the impact energy was 75 J. Applying higher impact energies of 150 J and 225 J showed that the absorbed energy in quasi-isotropic laminates is similar or lower than that found in cross-ply and angle ply laminates. This could be due to the larger failure strain of the glass fibre composites than that of carbon fibre composites since glass fibres have greater elongation.

**Figure 10:**
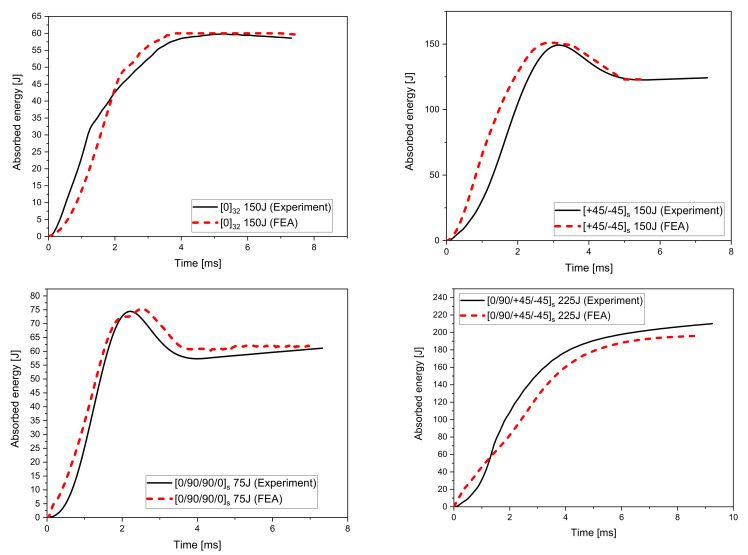
FE results for the absorbed energy vs. time curves for S2/FM94 glass fibre plates with different fibre orientation systems under 150 J impact energy.

The absorbed energy, as extracted from the FE model, is shown in Figure 10 for the selected energy of 150 J. The low absorption of the impact energy from the unidirectional plates is also demonstrated in the FE model, which denotes the well-predicted behaviour of the plates’ performance using the developed model.

**Figure 11:**
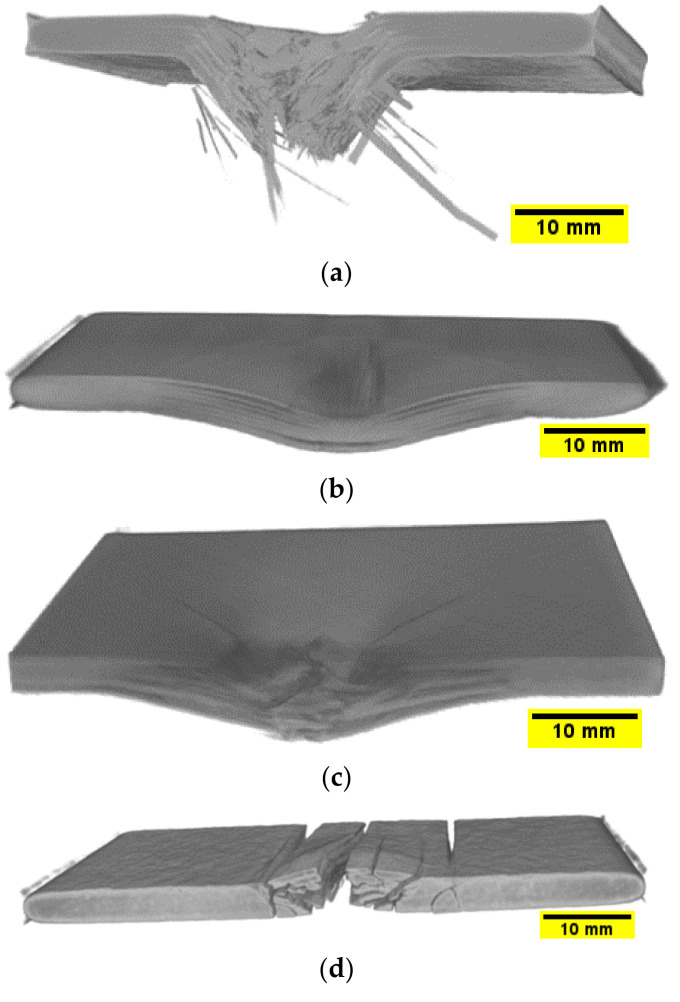
3D CT scan views of samples after impact using an energy of 225 J (**a**) ${\left[0/90/+45/-45\right]}_{8s}$ (**b**) ${\left[0/90/90/0\right]}_{8s}$ (**c**) ${\left[+45/-45\right]}_{16s}$ (**d**) $\;{\left[0\right]}_{32}$

**Figure 12:**
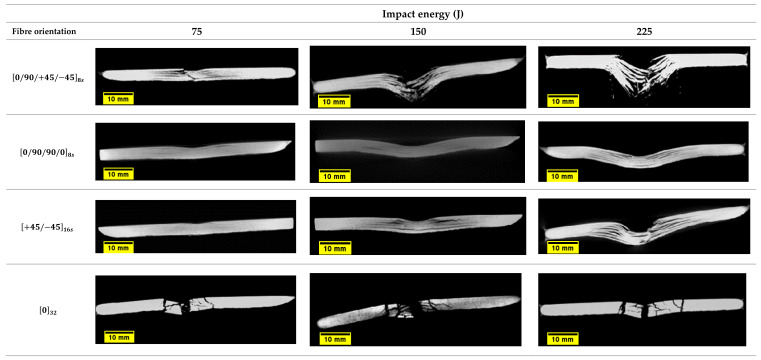
Cross-sectional micrographs of the S2/glass fibre plates impacted at different energy levels.

The damage type, location, and extent in the impacted plates were identified using computerised tomography (micro-CT scan). Figure 11 and Figure 12 show three dimensional and cross-sectional side views of the plates after impact. The visual and CT inspection of plate 4 ${\left[0\right]}_{32}$ at all energy levels show that they failed purely due to matrix cracking due to shear stresses characterised by shear, longitudinal, and transverse matrix cracking. For ${\left[0/90/90/0\right]}_{8s}\;$and ${\left[+45/-45\right]}_{16s\;}$plates, the dominating failure mechanism is fibre/matrix debonding, while for ${\left[0/90/+45/-45\right]}_{8s}\;$plates, the failure mechanism is a combination of shear matrix cracking and fibre/matrix debonding. Moreover, for ${\left[0/90/+45/-45\right]}_{8s}$ plates fibre fracture appears to occur even before any cracking in the off-axis plies takes place. It can also be observed that for quasi-isotropic plates, there appears to be a certain degree of interaction between cracks in certain adjacent plies (orientations). For example, it was previously reported that the 45° cracks promote enhanced cracking in the 90° plies [53], thus leading to further stiffness degradation in the laminate. As reported earlier in the literature, the stacking sequence in composites plays an important role in the impact resistance and resulting damage [2]. Generally, the energy in a composite laminate can be easily transferred from one ply to the other if they both have the same stacking sequence, and therefore, the resulting damage and the rate of crack propagation is greater than what would be if the composite layers have different stacking sequence. Therefore, a composite laminate having different fibre orientations will reduce the energy transfer through its thickness and will fail at a higher load [33]. Therefore, from a crack resistance point of view, a [0/90/0/90] stacking sequence is preferred over [0/0/90/90] or [0/90/90/0] [54]. This also supports the observation that no delamination is present in ${\left[0\right]}_{32}$ plates since delamination does not usually propagate between plies of the same orientation [55]. However, as evident from Figure 12, the interlaminar interface between laminates with different ply orientations is mechanically weak due to the mismatch in the bending deformations of adjacent plies [3]. Therefore, delamination occurs at the interfaces between glass fibre layers with different orientations, and the local separation from one another during impact is a common form of damage in such configurations [2,56], as can be seen in Figure 11a–c. Similar conclusions were reported by De Morais et al. [57], who observed that the impact resistance in non-symmetrical and cross-ply laminates was greater than in unidirectional laminates. They concluded that changing the stacking sequence in the laminate improved its mechanical properties and helped limit crack propagation at resin matrix dominated areas. This could also be attributed to the differences in bending stiffness of the laminates due to the stacking sequence. In another study, YiOu et al. [58] performed low-velocity impact tests on E-glass fibre reinforced FM94 epoxy resin prepreg. In their work, it was found that over 90% of the impact energy was absorbed by the bending/shear deformation which resulted in delamination and matrix cracking. In a similar manner, the cross-ply laminate ${\left[0/90/90/0\right]}_{8s}$ exhibited a greater impact resistance at 225 J as compared to the other three laminates in this study also shown in Figure 11b, with a high impact load at ~40 kN, as shown in Figure 8c. On the contrary, the damage mechanisms present in the ${\left[0/90/+45/-45\right]}_{8s}$ laminate involve high fibre breakage and pull-out especially in the ±45° fibre direction, which justifies the different bending stiffness of this laminate and the damage mechanisms that appear in Figure 11a.

.

**Figure 13:**
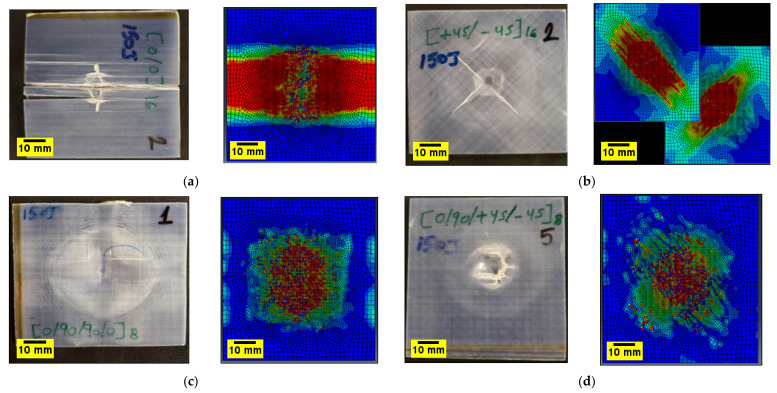
Comparison of damaged specimens (front side) after the impact at 150 J and FE model results for (**a**) ${\left[0\right]}_{32}$ (**b**) ${\left[+45/-45\right]}_{16s}$, (**c**) ${\left[0/90/90/0\right]}_{8s},$ and (**d**) ${\left[0/90/+45/-45\right]}_{8s}$ composite laminate.

Damage mechanisms observed in the impacted plates were primarily dominated by matrix cracks and delamination, which develop at an early stage of impact or contact with the samples at the top plies. They are caused by the transverse through-thickness tensile stresses due to the Hertzian contact effect, which caused the cracks to propagate in the thickness direction [59]. For ${\left[0/90/90/0\right]}_{8s}$ and ${\left[+45/-45\right]}_{16s}$ plates, in the inner interfaces, delamination in each ply is slightly away from the impact site. The delamination is elongated along the fibre direction of the layer, as is the case observed in ${\left[0/90/90/0\right]}_{8s}$, ${\left[+45/-45\right]}_{16s}$and ${\left[0/90/+45/-45\right]}_{8s}$plates. This was also observed in the FE results when the impact damage contours were compared with the experimental ones (Figure 13). The tendency of the damage mechanism is well predicted for all plates, as shown in Figure 13a–d for 150 J, where the fibre failure is shown in the damaged area. Several elements demonstrate fibre failure through the thickness of the laminates, shown as small, damaged elements in Figure 13. For the ${\left[+45/-45\right]}_{16s}$, plates the cracks initiate along the direction of the fibres, a phenomenon that is depicted for the top two laminates of the FE results in Figure 13b.

**Figure 14:**
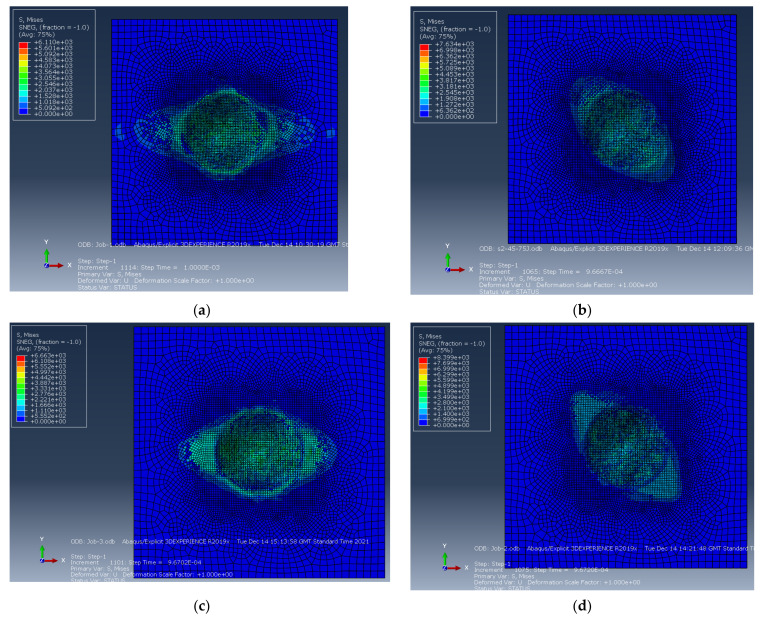
Numerical prediction of delamination damage at the top surface of (**a**) ${\left[0\right]}_{32}$ (**b**) ${\left[+45/-45\right]}_{16s\;}$ (**c**) ${\left[0/90/90/0\right]}_{8s},$ and (**d**) ${\left[0/90/+45/-45\right]}_{8s}\;$ composite laminate.

Additionally, the predicted damaged area for all the laminates is shown in Figure 14 for the 75 J impact energy. Reasonably good agreement is achieved in predicting the location and extent of damage by the FE model, the overall damage area and in terms of the delamination mapping using the cohesive contact, which appears to be able to simulate the composite laminate delamination onset and propagation caused by low-velocity impact.

**Figure 15:**
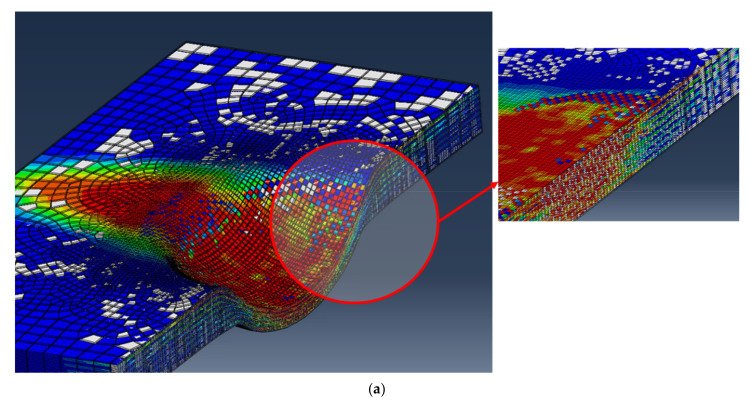

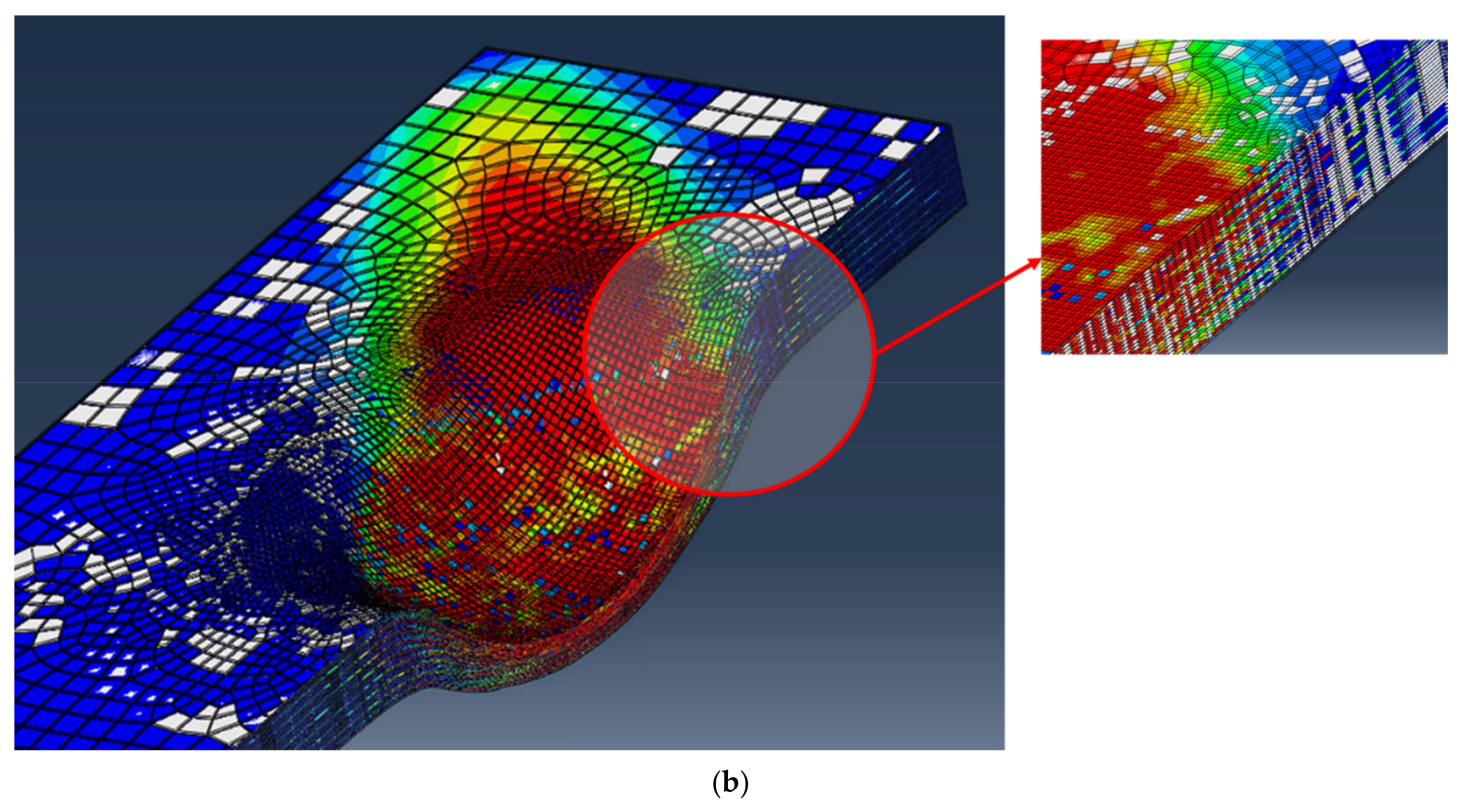
Damage contour with delamination through the thickness of the cross-section of the (**a**) ${\left[0/90/90/0\right]}_{8s},$ and (**b**) ${\left[0/90/+45/-45\right]}_{8s}$ plates.

The impact cone region at the centre of the samples where the impactor is in full contact with the samples is known as the impact compression zone by previous researchers [59,60,61,62]. This region is under low shear stresses and is characterised by through-thickness compression; therefore, the onset of delamination does not initiate from there [60]. The brittle nature of glass fibres and the high impact energy used prevents the generation of impact compression, and therefore, samples undergo full penetration, as is the case for the ${\left[0/90/+45/-45\right]}_{8s}$ plates at 150 J and 225 J. For ${\left[+45/-45\right]}_{16s\;}$ and ${\left[0/90/+45/-45\right]}_{8s}\;$plates, it can be seen that the impact energies of 150 and 225 J are above the critical impact level which they can withstand; ply separation followed by partial or complete perforation is observed. The ply separation is followed by interface delamination which spreads away from the centre of the samples; this is a strong indication that delamination occurs between plies having different fibre orientations. Moreover, the delamination between plies tends to elongate along the fibre direction of the lower layer at the interface, with the largest delamination appear to be developing between layers with the greatest mismatch in fibre orientation [60,63]. For ${\left[0/90/90/0\right]}_{8s}$, ${\left[+45/-45\right]}_{16s\;},$ and ${\left[0/90/+45/-45\right]}_{8s}\;$plates, the delamination across plies appears to have regular pattern characterised by a three-dimensional spiral staircase as reported by Abrate et al. [6]. Therefore, it can be said the total thickness of unidirectional layers with same fibre orientation in laminates with dispersed stacking sequence are thinner and have more interfaces both of which increase the likelihood of delamination [55]. The onset and propagation of interlaminar delamination between differently or similarly oriented plies is measured from the FE model and presented in Figure 14. In this figure, a selected representation of the damaged area for the ${\left[0/90/90/0\right]}_{8s}$ and the ${\left[0/90/+45/-45\right]}_{8s}$ plates at 150 J are given. These two plates were chosen because of the complexity of their plies orientation to demonstrate how the delamination evolves and propagates through differently oriented plies. By comparing the contours of Figure 15, it is evident that the delamination is present between plies with different fibre orientations, whereas when the 0° or the 90° plies are adjacent, the delamination did not change (Figure 15a). The use of cohesive elements between all plies through the thickness of the plate has proven very efficient to capture this behaviour. It is well known that multidirectional laminates possess higher interlaminar fracture resistance to impact loading due to increased toughening mechanisms [64]. For ${\left[0/90/90/0\right]}_{8s}$ and ${\left[+45/-45\right]}_{16s}$ plates, no full penetration occurs, while ${\left[0/90/+45/-45\right]}_{8s}$ undergoes full penetration, as shown previously in Figure 12. The multidirectional stacking sequence in those samples can help prevent matrix crack propagation through thickness due to the increased fibre bridging effect [55].

Quadriaxial fabrics are quasi-isotropic and the layup does provide strength in the four fibre directions, nevertheless, their mechanical response depends on the number plies having the same fibre orientation through-thickness. This, in return, weakens through-thickness resistance to compressive loadings and limits their interlaminar fracture resistance. Indeed, Fouss et al. [5,65] recommend avoiding ply clustering despite it improving the deflectional stiffness; it weakens the structure of composite as fewer through-thickness interfaces are available to absorb the energy of the impact. Therefore, it can be said that the impact response of S2/glass fibre plates can be improved through the design of composite laminates and finding the multiple stack optima. However, this does not mean that having a vastly dispersed stacking sequence would imply better impact resistance, but rather that there is more than one stacking sequence that satisfies a given design criterion (i.e., improved impact resistance) [55].

## 5. Conclusions

In this study, low-velocity impact tests were carried out to evaluate the effect of ply orientation and impact energy level on the behaviour and induced damage in glass fibre composite plates using three energy levels. The composite plates were made using unidirectional S2/FM94 glass fibre epoxy layers with four different staking sequences, namely $\;{\left[0\right]}_{32}$, ${\left[0/90/90/0\right]}_{8s}$, ${\left[0/90/+45/-45\right]}_{8s},$ and ${\left[+45/-45\right]}_{16s}$. A finite element model was developed using Abaqus/SIMULIA software to predict the impact energy and onset of failure, and the results were compared against the experimental data to validate the accuracy of the model. In addition, an X-ray computed tomography scan was employed to study the visible and internal damage mechanisms which occurred in the impacted plates. The results were discussed to relate the effect of ply orientation on the impact damage characteristics such as impact load capability and absorbed energy. The following results can be concluded from the study:

- Plates fabricated using$\;{\left[0\right]}_{32\;}$fibre orientation was the least resistant to impact at all tested energy levels. The samples were severely damaged and failed purely due to shear stresses without delaminating.
- The impact tests showed that the plates with ${\left[0/90/90/0\right]}_{8s}$ configuration absorbed more energy with less penetration depth than the plates with other stacking configurations.
- CT scans revealed that delamination was the main failure mechanism in plates fabricated using ${\left[0/90/90/0\right]}_{8s}$, ${\left[+45/-45\right]}_{16s}$, and ${\left[0/90/+45/-45\right]}_{8s}$ orientation.
- Finite element models showed good agreement with experimental data and accurately predicted the failure modes in the plates due to impact.
- The use of cohesive elements between each ply of the laminate is a very useful technique to capture the delamination between differently oriented plies, but also to prove that the delamination onset occurs first when the fibres have different orientations between adjacent plies.
- From the four different plates tested, it was found that plates with ${\left[0/90/90/0\right]}_{8s}$ stacking sequence showed better performance under the impact, whereas the unidirectional plates showed poor performance at all energy levels and were comparable to the ${\left[0/90/+45/-45\right]}_{8s}$ plates. The ${\left[+45/-45\right]}_{16s}$ performed satisfactorily at low and medium energy, which makes it the second suitable candidate for the studied loading condition.
- The presented findings in this study incorporating experimental and validated numerical modelling will help researchers in aerospace engineering in understanding the influence of various important parameters on the impact damage characteristics of S2/FM94 glass/epoxy composite laminates to be used in aircraft structures.

## Figures and Tables

**Table 1:** Ply configuration of the four plates used in the impact study.

**Plate Number**	Plate 1	Plate 2	Plate 3	Plate 4
**Ply orientation**	${\left[0/90/+45/-45\right]}_{8s}$	${\left[0/90/90/0\right]}_{8s}$	${\left[+45/-45\right]}_{16s}$	$\;{\left[0\right]}_{32}$

**Table 2:** Impact energy levels used in the study.

Impact Level	Level 1	Level 2	Level 3
**Impact Energy (J)**	75	150	225

**Table 3:** Strength properties of S2 Glass/FM 94 epoxy prepreg.

$X_{T}$ $\left(MPa\right)$	$X_{C}$ $\left(MPa\right)$	$Y_{T}$ $\left(MPa\right)$	$Y_{C}$ $\left(MPa\right)$	$S_{L}$ $\left(MPa\right)$	$S_{T}$ $\left(MPa\right)$
2430	2000	50	150	76	50

**Table 4:** Material data of S2-FM94 lamina used in the FE analysis.

Density (Kg/m^3^)	*E*_11_ (GPa)	*E*_22_ (GPa)	*E*_33_ (GPa)	*G*_12_ (GPa)	*G*_13_ (GPa)	*G*_23_ (GPa)	*ν*_12_	*ν*_13_	*ν*_23_
1980	53.98	9.412	9.412	5.548	3	5.548	0.0575	0.0575	0.33

**Table 5:** Fracture energies for fibre and matrix tension and compression failure modes for S2-FM94 glass fibre adhesive epoxy.

$G_{ft}^{C}$ **(kJ/m^2^)**	$G_{fc}^{C}\;(\mathrm{kJ}/m^{2})$	$G_{mt}^{C}\;(\mathrm{kJ}/m^{2})$	$G_{mc}^{C}\;(\mathrm{kJ}/m^{2})$
12.5	12.5	1.0	1.0

**Table 6:** Inter-laminar strength and fracture energies of cohesive interfaces of FM94 adhesive.

$t_{n}^{0}$ $\left(MPa\right)$	$t_{s}^{0}$ $\left(MPa\right)$	$t_{t}^{0}$ $\left(MPa\right)$	$G_{n}$ (kJ/m^2^)	$G_{s}$ (kJ/m^2^)	$G_{t}$ (kJ/m^2^)	*E/E*_nn_ (GPa/mm)	*G*_1_*E*_*ss*_ (GPa/mm)	G_2_*E*_*tt*_ (GPa/mm)
50	50	60	4.0	4.0	4.0	10^5^	10^5^	10^5^

**Table 7:** FE model setup for impactor and plate.

Setup	Impactor	Plate
**Mesh size**	2 × 2 mm^2^	1 × 1 mm^2^
**Element type**	R3D3, R3D4	SC8R, COH3D8
**Number of elements**	1431	475,272
**Material**	Steel	S2 glass fibre composite
**Body type**	Discrete rigid non-deformable	Deformable
**Solver**	Dynamic Explicit	

## Data Availability

Data can be provided upon contacting the first author.
